# Optical Properties of Cationic Perylenediimide Nanowires in Aqueous Medium: Experimental and Computational Studies

**DOI:** 10.1007/s10895-023-03253-9

**Published:** 2023-06-06

**Authors:** Ahmed M. Kobaisy, Marwa F. Elkady, Ahmed A. Abdel-Moneim, Erol Yildirim, Ahmed El-Shafei, Mohamed E. El-Khouly

**Affiliations:** 1 Nanoscience Program, Institute of Basic and Applied Science, Egypt-Japan University of Science and Technology (E-JUST), New Borg El-Arab City, Alexandria, Egypt; 2 Chemical and Petrochemicals Engineering Department, Egypt-Japan University of Science and Technology (E-JUST), New Borg El-Arab City, Alexandria, Egypt; 3 Fiber and Polymer Science Program, North Carolina State University Raleigh, Raleigh, NC 276060 USA

**Keywords:** Perylenediimide, Optical materials, Light harvesting, Electron transfer

## Abstract

Cationic perylenediimide derivative, namely N,N’-di(2-(trimethylammoniumiodide)ethylene) perylenediimide (TAIPDI), has been synthesized and characterized in an aqueous medium by using dynamic light scattering (DLS), X-ray diffraction (XRD), fourier-transform infrared (FTIR), scanning electron microscope (SEM), and high-resolution transmission electron microscopy (HRTEM) techniques. The optical absorption and fluorescence spectra of TAIPDI revealed the formation of aggregated TAIPDI nanowires in water, but not in organic solvents. In order to control the aggregation behavior, the optical properties of TAIPDI have been examined in different aqueous media, namely cetyltrimethylammonium bromide (CTAB), and sodium dodecyl sulfate (SDS). Furthermore, the utilization of the examined TAIPDI for constructing supramolecular donor–acceptor dyad has been achieved by combining the electron accepting TAIPDI with the electron donating 4,4’–bis (2-sulfostyryl)-biphenyl disodium salt (BSSBP). The formed supramolecular dyad TAIPDI-BSSBP through the ionic and electrostatic π-π interactions have been well examined by various spectroscopic techniques, e.g., steady-state absorption and fluorescence, cyclic voltammetry, and time-correlated single-photon counting (TCSPC), and first principle computational chemistry methods. Experimental results suggested the occurring of intra-supramolecular electron transfer from BSSBP to TAIPDI with rate constant and efficiency of 4.76 × 10^9^ s^−1^ and 0.95, respectively. The ease of construction, absorption in the UV–Visible region, and fast electron transfer process render the supramolecular TAIPDI-BSSBP complex as a donor–acceptor material for optoelectronic devices.

**Supplementary Information:**

The online version contains supplementary material available at 10.1007/s10895-023-03253-9.

## Introduction

With rapidly growing global energy consumption, the world faces serious energy, environmental, and economic crises as a result of depleted stocks of fossil fuels, pollution, climate change, etc. Prompt global action to solve the energy crisis is an urgent need. To pursue such an action, the world must develop sources of energy that are affordable, accessible, clean, and sustainable from economic prospects [1–9]. Toward this goal, various functional dyes have been employed for converting energy the light energy into a stable electrochemical energy [10–15]. Among them, n-type organic semiconductor perylenediimides (PDIs) are considered promising electron-accepting materials for their unique optical and electrical properties, e.g., the capacity to absorb light over a substantial part of the visible spectral region, strong electron affinities, appropriate redox values, excellent chemical and photochemical stability, significant charge transport properties, and strong fluorescence emission [16–24]. These unique photophysical, photochemical, and electrochemical properties render PDIs potential candidates for various optoelectronic applications [25–27]. The fact that PDIs exhibit a first reduction potential comparable to that of fullerene C_60_ render PDIs attractive acceptors for replacing fullerene derivatives in photovoltaic applications with their relatively lower cost in comparison to C_60_-based acceptors, as well as better light harvesting and ease of chemical modification [16–24].

The ability of perylenediimides to form supramolecular donor–acceptor light-harvesting architectures by π-π-stacking, ionic bonding, hydrogen bonding, and metal-ion coordination [28–34] justifies their wide use as building blocks for the construction of light harvesting complexes. Accordingly, perylenediimides have been incorporated successfully into a variety of self-assembled light-harvesting architectures in organic solvents [15–21, 35–37], but not in aqueous media.

**Fig. 1:**
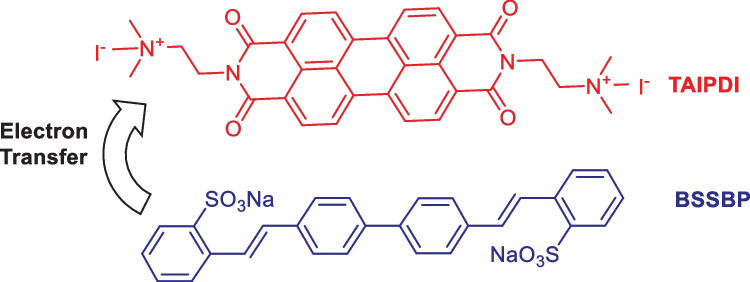
Molecular structure of the examined TAIPDI-BSSBP supramolecular dyad

To examine the optical behavior of PDIs in an aqueous medium, we utilized herein water soluble perylenediimide derivative, namely N, N′-bis (2-(trimethylammonium iodide) ethylene) perylenediimide (TAIPDI) (Fig. 1). Due to its excellent electron-accepting properties, the ability of TAIPDI to form supramolecular donor–acceptor complex has been examined by combining it with the electron-donating stilbene 420 dye, which is well known as 4,4’–bis (2-sulfostyryl)-biphenyl disodium salt (BSSBP). The rationale for choosing the TAIPDI-BSSBP supramolecular dyad and the design concepts are:

- TAIPDI and BSSBP are linked together to form from the supramolecular TAIPDI-BSSBP dyad through π-π and ionic and electrostatic π-π interactions.
- TAIPDI-BSSBP supramolecular dyad, owing to the presence of TAIPDI and BSSBP units that absorb light in a wide range of the solar spectrum (200–600 nm), guarantees an increased absorption cross-section and an efficient use for converting the light to chemical energy.
- A practical aspect of BSSBP and TAIPDI concerns their highly fluorescence quantum yields in the organic solvents, serving diagnostic probes for the intramolecular electron transfer event in the supramolecular dyad.

The optical behavior of TAIPDI, as well as the electron transfer character of the supramolecular complex (TAIPDI-BSSBP) in an aqueous medium have been examined using various spectroscopic techniques, e.g., steady-state absorption and fluorescence, and time-correlated single-photon counting, and cyclic voltammetry.

## Experimental Section

### Materials

Disodium-4, 4’-bis(2-sulfonatostyryl) biphenyl (BSSBP) is commercially available and purchased from Tokyo Chemical Industry (TCI). Milli-Q system (Millipore) was used for water purification to obtain ultrapure water (18.2 M $\Omega$ cm) for the experiments. Sodium n-dodecyl sulfate (SDS, 99%, dry water < 15%) and cetyltrimethylammonium bromide (CTAB) were purchased from Aldrich and used without any further purification. All organic solvents were from Aldrich and used as received.

**Scheme 1:**
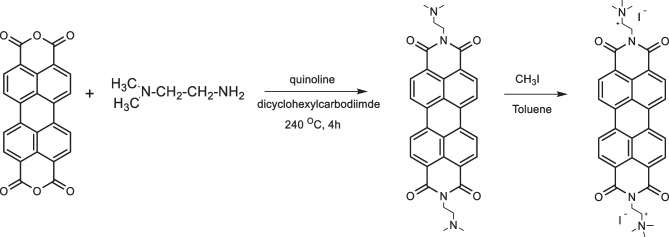
Schematic representation of the synthetic route to TAIPDI

N, N’-bis(2-(trimethylammoniumiodide) ethylene) perylenediimide (TAIPDI) was synthesized according to the reported procedure [22]. In a typical procedure shown in Scheme 1, perylenetetracarboxylic-3,4,9,10- bisanhydride (1 g, 2.5 mmol), quinoline 15 ml, 2-N,N′-dimethylaminoethylenediamine (0.2 g, 20 mmol) and dicyclohexylcarbodiimide (0.35 g, 1.7 mmol) were refluxed with stirring in an inert nitrogen atmosphere at 230–240 °C for 3–4 h. After cooling to room temperature, the reaction mixture was poured into 200 ml of ethanol. The resultant precipitate was filtered off and dried. Methyl iodide (20 ml, 5 mmol) in toluene (80 ml) was added and the product PDI (2.5 mmol) was refluxed with vigorous stirring for 90 min. After cooling to room temperature, the resultant precipitate was filtered off, washed with ether, and dried in a vacuum to afford TAIPDI (1.45 g). Fig. S1 shows the structural characterization of TAIPDI by different techniques. ^1^H-NMR (400 MHz, DMSO-d6, TMS): *δ* = 3.28–3.34 (m, 18H), 3.67–3.71 (t, 4H), 4.49–4.53 (t, 4H), 8.45–8.47 (d, 4H, 8.72–8.74 (d, 4H); ^13^C NMR (400 MHz, DMSO-d6, TMS): *δ* = 33.6, 52.4, 61.7, 122.2, 124.1, 125.1, 128.2, 130.7, 133.7, 162.5 ppm. FT-IR (KBr, cm^–1^): *ν* = 3551, 3414, 3009, 1696, 1649, 1593, 1480, 1437, 1434, 1366, 1365, 1259, 1182, 1118, 1042, 998, 927, 848, 811, 745, 628.

### Instruments

The synthesized TAIPDI was identified utilizing ^1^H and ^13^C NMR spectroscopy (AL 400, JEOL Ltd., Tokyo, Japan). The compounds were purified by size exclusion chromatography (column: Shodex K-5001, eluent: CHCl_3_, Showa Denko K. K., Tokyo, Japan). Fourier transform-infrared (FT-IR) spectra were measured using FT-IR spectrometry (FT/IR-4100, Jasco). Self-assembly of TAIPDI was investigated using X-ray diffractometry (XRD; Smart Lab, Rigaku, Tokyo, Japan). Particle sizes were determined using a particle size analyzer (ELSZ-2PL, Otsuka Electronics Co. Ltd., Osaka, Japan) in water. Steady-state absorption and fluorescence spectra were recorded using UV–vis spectrophotometer (Shimadzu UV-2600) and spectrofluorophotometer (Shimadzu RF-6000), respectively. To elucidate the electrochemical properties and to determine the energy of the electron transfer product (TAIPDI^**.**−^-BSSBP^**.**+^), a Metrohm Autolab Potentiostat/Galvanostat electrochemical analyzer in deionized water containing Na_2_SO_4_ (0.1 M) as a supporting electrolyte at 298 K has been utilized. The measurements were taken by using a carbon electrode as a working electrode, an Ag/AgCl as a reference electrode, and a platinum wire (Pt, 1 mm in diameter) as a counter electrode. All electrochemical measurements were investigated under a nitrogen atmosphere. For determining the electron transfer character and determine the electron-transfer rate of the TAIPDI-BSSBP supramolecular dyad, the picosecond fluorescence decay profiles were measured by the single-photon counting method using FluoroHub (Horiba Scientific). Lifetime was evaluated with the software Fluofit attached to the equipment.

## Results and Discussion

### Optical Properties and Aggregation Behavior of One-Dimensional TAIPDI

**Fig. 2:**
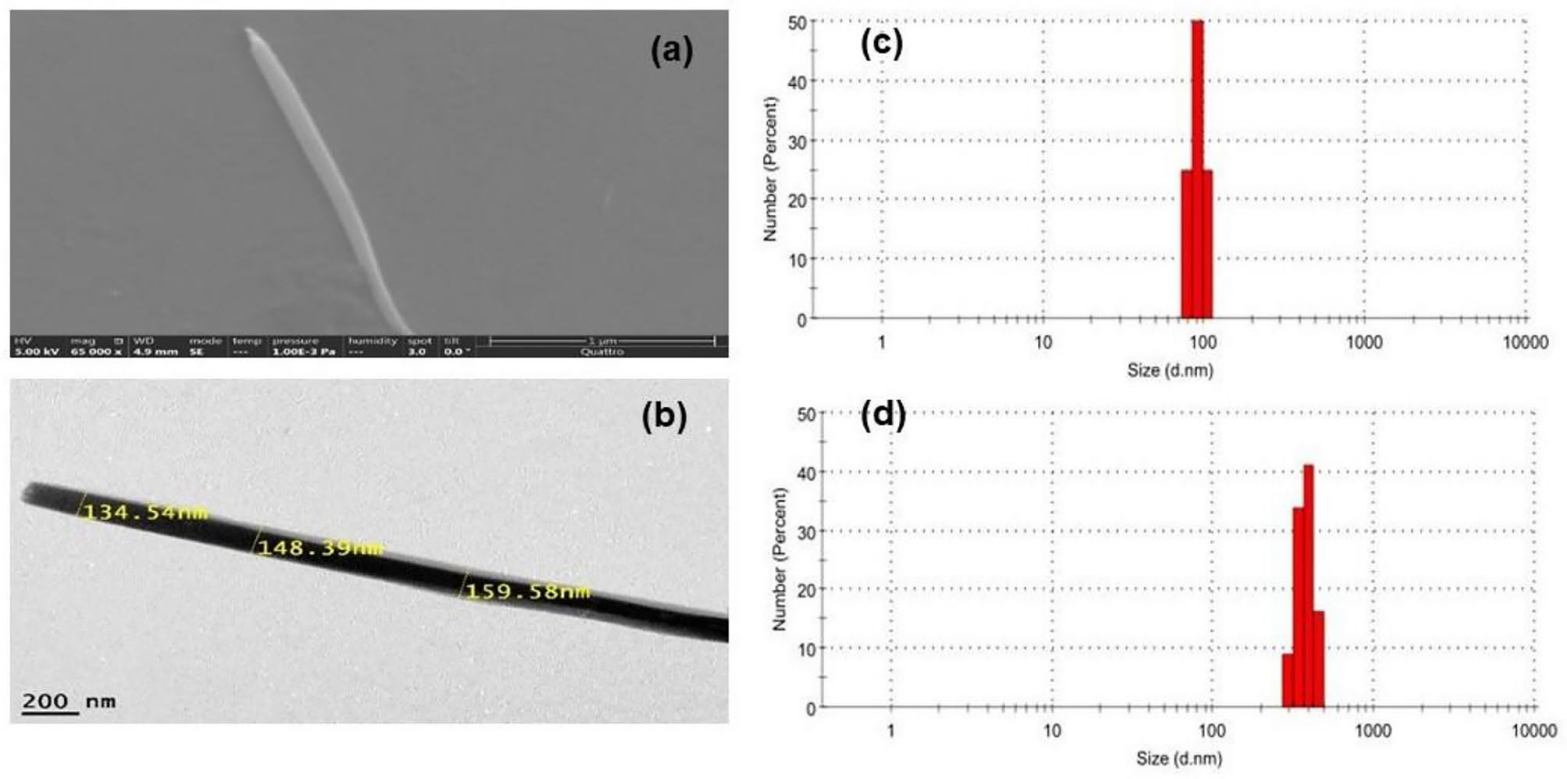
**a** SEM and **b** HR-TEM images of aggregated TAIPDI nanowire in water, **c** and **d** DLS particle size distribution of TAIPDI in methanol, and water, respectively

As seen from Fig. 2, SEM and HR-TEM images of TAIPDI molecules show ordered nanowire assemblies with an average width of approximately 200–300 nm and lengths up to tens of micrometers. This is consistent with the literature, where the perylenediimide derivatives with linear side chains and extensive π-stacking reveal one-dimensional morphologies in aggregation [22]. The recorded narrow average width of TAIPDI nanowires (200–300 nm) may arise from the electrostatic repulsion between the positively charged trimethylammonium heads. By using the dynamic light scattering (DLS) technique, we could identify the size distribution of the self-assembled TAIPDI in water in the range of 300–400 nm, with a mean size of 379 nm (Fig. 2d) that is much higher than that of the TAIPDI in MeOH (91 nm). This finding suggests the formation of aggregated TAIPDI in water, but not in MeOH.

**Fig. 3:**
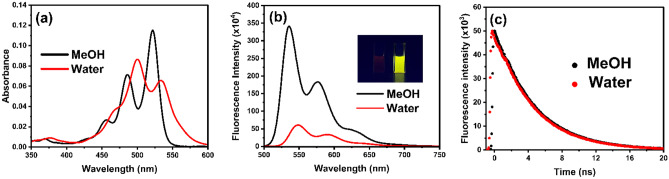
**a** Absorption spectra of TAIPDI (1.5 µM) in water and methanol. **b** Fluorescence spectra of TAIPDI (1.5 µM) in water and methanol; λ_ex_ = 480 nm. Inset: Photograph of TAIPDI solution in water (left) and methanol (right) under lamp irradiation; λ_ex_ = 365 nm. **c** Fluorescence decay profiles of TAIPDI (1.5 µM) in water and methanol; (λ_ex_ = 372 nm, λ_ex_ = 550 nm)

As seen in Fig. 3a, the absorption spectrum of TAIPDI in MeOH exhibited three pronounced peaks at 522, 487, and 456 nm as representative 0–0, 0–1, and 0–2 transitions, respectively. Similar optical behavior was observed in dimethylformamide and acetone (Fig. S2). This observation is in a good agreement with the multiple vibronic bands of the reported perylenediimide derivatives in organic solvents [19–23]. Unlike methanol, the absorption spectrum of TAIPDI in water showed different features, where the absorption spectrum exhibited two absorption maxima at 501 and 536 nm, as well as a broad shoulder at approximately 467 nm. As seen, the ratio of A_(0–0)_/A_(0–1)_ for the two main absorption bands of TAIPDI in water (A_536 nm_/A_501 nm_ = 0.60) is much smaller than that observed in MeOH (A_522 nm_/A_487 nm_ = 1.60). Taking into consideration that the aggregation of PDIs occurs when the absorption ratio of A_(0–0)_ /A_(0–1)_ < 0.7 [35–37], we can attribute the absorption behavior of TAIPDI in water is due to of the formation of aggregated TAIPDI in H_2_O via strong π-π interactions of hydrophobic aromatic cores [36–39].

On the other hand, the optical behavior of TAIPDI was examined by using the steady-state emission technique (Fig. 3b). Upon irradiation with 450 nm exiting light, the fluorescence spectrum of TAIPDI in MeOH exhibited two strong emission bands at 538 and 577 nm and a shoulder at 628 nm with a fluorescence quantum yield (ɸ_f_) of 0.7. Unlike MeOH, the fluorescence spectrum of TAIPDI in water exhibited weak emission bands at 550 and 591 nm with a fluorescence quantum yield of 0.11. Such change in the fluorescence pattern in water and the small fluorescence quantum yield of TAIPDI confirm the aggregated TAIPDI in water. It should be noted the inner filter effects on the fluorescence quenching was excluded considering that the absorbance value at the excitation wavelength is quite low (nearly 0.06).

**Fig. 4:**
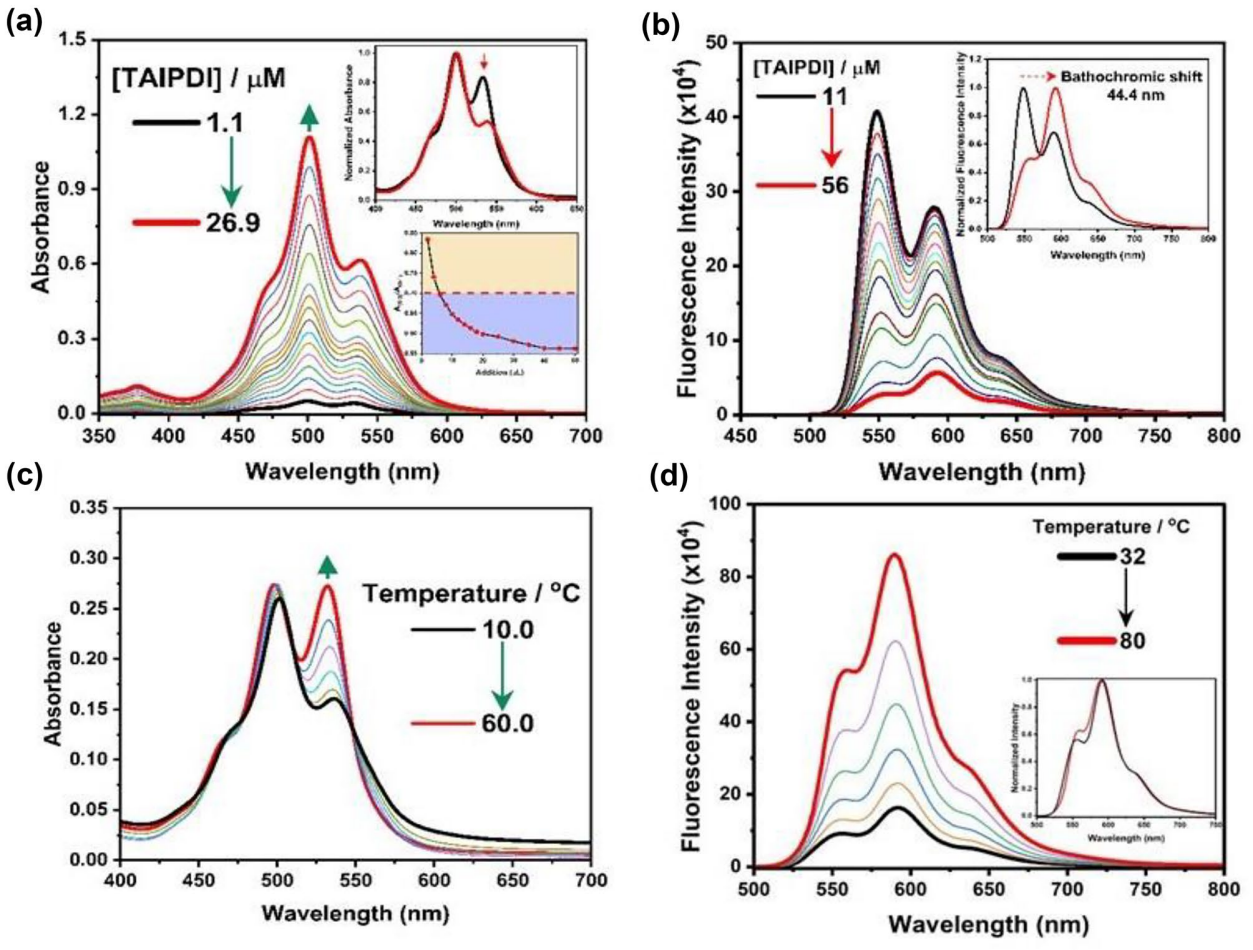
**a** Steady-state absorption spectra of TAIPDI (1.1–26.9 µM) in water (Inset: graph showing the ratio between A_(0–0)_/A_(0–1)_ upon increasing the concentration of TAIPDI), **b** Fluorescence spectra of different concentrations of TAIPDI (11–56 µM); λ_ex_ = 480 nm (Inset: Normalized fluorescence spectra for the start and the end concentrations to clarify the bathochromic shift and the change in peak ratios), **c** Absorption spectra of TAIPDI (10.92 µM) with increasing the temperatures from 10 ºC to 60 ºC, **d** Fluorescence spectra of TAIPDI (10.92 µM) with increasing the temperatures from 32 ºC to 80 ºC

Fluorescence lifetime measurements (TCSPC) for the same concentrations of TAIPDI in water and methanol track the same consideration as in the steady-state fluorescence measurements (Fig. 4c). However, the data could be fitted as mono-exponential decay with a fluorescence lifetime value of 4.68 ns in methanol. The finding of the fluorescence lifetime in water (4.75 ns) is very close to that of the monomer form in MeOH, may suggested that the TAIPDI aggregates in water to produce non-emissive species. With formation of the aggregated form in water, there is a still fraction of non-aggregated TAIPDI in the micromolar range, from where the residual emission was observed.

Furthermore, the formation of aggregated TAIPDI in water was confirmed by the effect of concentrations and temperatures as shown below:

(i) As shown in Fig. 4a, the absorption intensity of TAIPDI in water increases with increasing the concentrations of TAIPDI from 1 to 27 μM. The fluorescence intensity of TAIPDI exhibited maxima at 549 and 590 nm (Fig. 4b). With increasing its concentrations up to 27 μM, it was found that the fluorescence intensity was significantly decreased accompanied by a change of the relative intensity of the two emission bands (Fig. 4b). Such significant decrease of the fluorescence intensity with increasing [TAIPDI] indicates the formation of aggregated TAIPDI in water. Obviously, as TAIPDI concentration in water increases, the less monomers remain non-aggregated, which results in a clear decrease in fluorescence emission as the aggregates are completely non-emissive. When the concentrations of TAIPDI become higher, inner filter effects become more pronounced, which explain why the emission spectrum of the residual non-aggregated monomers become highly distorted (Fig. 4b). Such emission changes with increasing the concentrations may also affect the 0–0 vibronic emission band that the overlaps with the absorption spectrum and therefore, are indicative of self-absorption processes.
(ii) The absorption spectra of TAIPDI showed different features with increasing the temperatures as seen in Fig. 4c. The absorption ratio of TAIPDI at 501/536 nm was found to be 0.64, which is significantly increase to 0.99 with increasing the temperatures from 10 to 60 °C. Similarly, the fluorescence spectra of TAIPDI increase with increasing the temperatures from 32 °C to 80 °C (Fig. 4d). such findings also confirm the conversion of the aggregated TAIPDI into monomer TAIPDI with increasing the temperatures [36–39].

### Effect of Surfactants on Controlling the Molecular Aggregation of TAIPDI

**Fig. 5:**
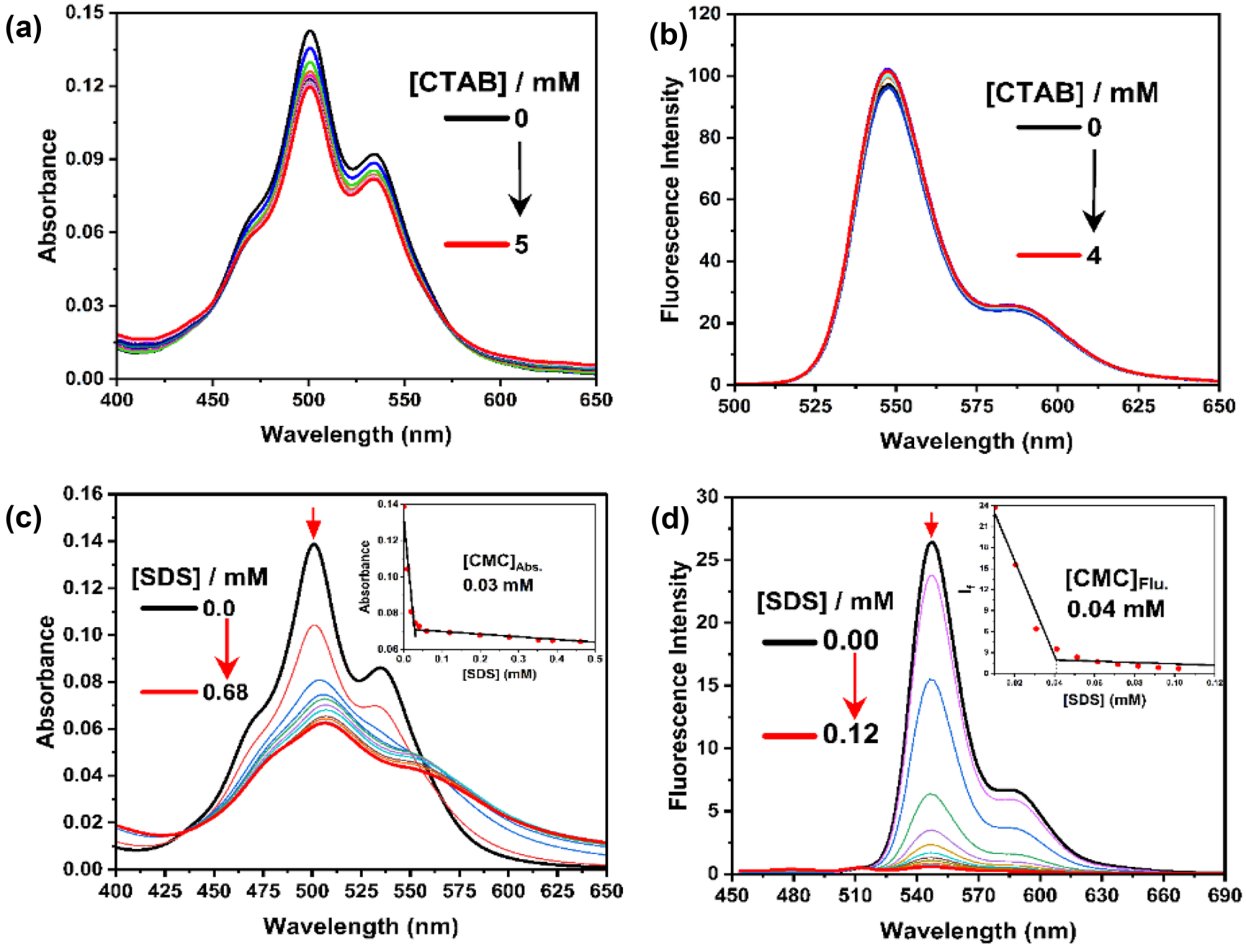
**a** Steady-state absorption spectra of TAIPDI [3.50 µM] with the addition of cetyltrimethylammonium bromide CTAB (0—5 mM) in water, **b** Fluorescence spectra of TAIPDI with the addition of CTAB (0—4 mM) in water; λ_ex_ = 459 nm, **c** Absorption spectra of TAIPDI [3.50 μM] in the presence of different concentrations of SDS, **d** Emission spectra of TAIPDI in the presence of different concentrations of SDS; λ_ex_ = 434 nm

**Fig. 6:**
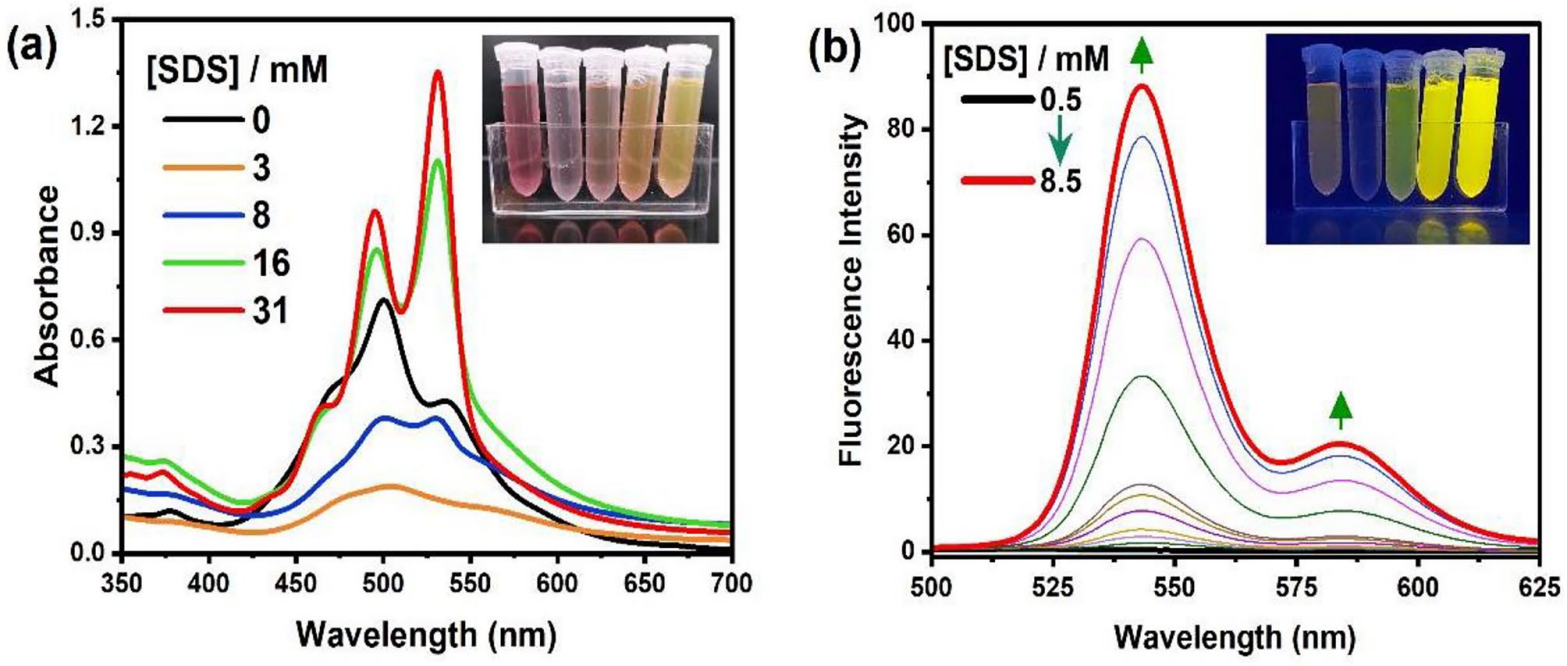
**a** Steady-state absorption spectra of TAIPDI before and after the addition of SDS (0–31 mM) in water; the inset shows the Image of TAIPDI aqueous solutions upon adding different concentrations of SDS (0.0, 3.1, 8.0, 15.5, 31.0 mM) from left to right, respectively, **b** Fluorescence spectra of TAIPDI in the presence of higher SDS concentrations (0.5—8.5 mM) of SDS at λ_ex_ = 434 nm, (Inset) TAIPDI aqueous solutions with different SDS concentrations (0.0, 3.1, 8.0, 15.5, 31.0 mM) from left to right respectively under UV lamp; *λ*_ex_ = 365 nm

Upon adding different amounts of cetyltrimethylammonium bromide (CTAB) to an aqueous solution of TAIPDI, we could not identify any significant change in both the absorption and fluorescence spectra of TAIPDI (Fig. 5). This observation indicates small effect for the aggregation of TAIPDI in the presence of cationic CTAB surfactants (Fig. 5a, b). On the other hand, the effect of anionic surfactant sodium dodecyl sulfate (SDS) showed different features. Upon adding different amounts of SDS to an aqueous solution of TAIPDI, we could notice a gradual decrease of the absorption bands at 501 and 535 nm accompanied by a red-shift to the main absorption band, suggesting the formation of TAIPDI aggregates in the presence of concentrations of SDS. From the inset Fig. 6c, the critical micelle concentration (CMC) value was determined to be 0.03 mM [40, 41]. Similarly, upon adding (0–0.12 mM) of SDS to an aqueous solution of TAIPDI, one could notice a significant fluorescence quenching of TAIPDI at 548 and 585 nm due to the increase the aggregated TAIPDI molecules with increasing [SDS] micelles. Such fluorescence quenching can be explained by the charge neutralization resulting from the interaction of anionic premicellar units of SDS and the dicationic TAIPDI and leading to the formation of non-fluorescent aggregates. From the plot of fluorescence intensity versus SDS concentration, we could mark the CMC value as 0.04 mM that is quite close to that recorded by absorption measurements.

Interestingly, with adding further amounts of SDS (above the CMC value) to a solution of TAIPDI in water, we observed a considerable increase in the A_(0–0)_/A_(0–1)_ ratio from 0.63 to 1.55, accompanied with ~ 30 nm red shift in the main absorption band (Fig. 6a). Similarly, the fluorescence spectra show a gradual increase in the fluorescence intensity accompanied by a 4-nm blue shift with increasing the concentrations of SDS up to 8.2 mM (Fig. 6b). These significant changes in the absorption and fluorescence spectra of TAIPDI in the presence of relatively high concentrations of SDS (above the CMC value) is also accompanied with a color change from red color to fluorescent yellow color. These observations indicate that the higher concentrations of SDS induce the fluorescence of TAIPDI by capturing it in the monomer form. Tang et al. reported a similar behavior upon adding DTAC (cationic surfactant) to an anionic PDI [41]. This can be explained as after CMC, the concentration of micelles increases, which facilitates capturing the neutralized TAIPDI in the monomeric form inside the formed micelles (Fig. S3).

### Effect of pH

**Fig. 7:**
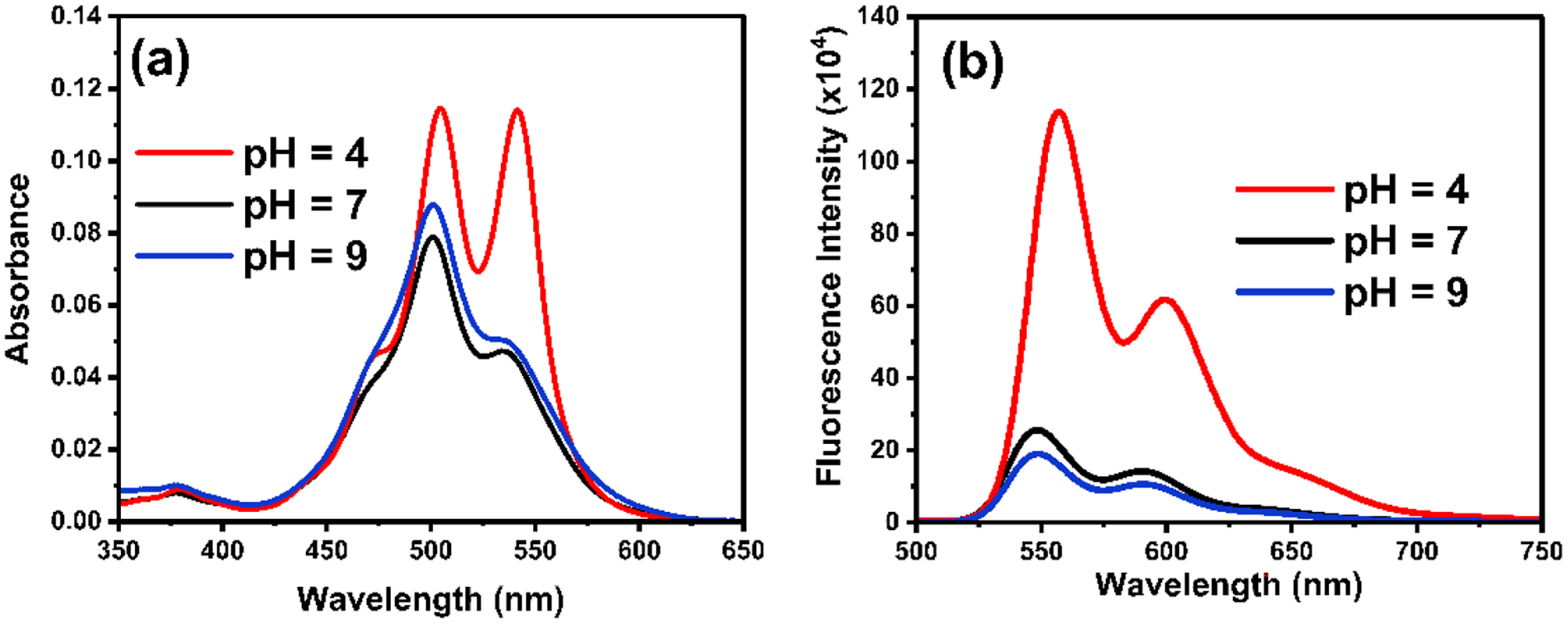
**a** Steady-state absorption spectra, **b** Fluorescence spectra of TAIPDI (1.1 µM) at different values of pH (4, 7, and 9); at λ_ex_ = 480 nm

The absorption spectrum of TAIPDI (1.1 µM) was studied using different pH buffers at pH values of 4, 7, and 9. The absorption spectrum at (pH = 4) (Fig. 7a) show absorption maximum at 504.5 nm representing the 0–1 transition along with another sharp peak at 542 nm with slightly lower absorbance representing the 0–0 transition representing lower aggregation. In neutral buffer solution (pH = 7), we can observe an absorption maximum at 501 nm representing the 0–1 transition. Also, a noticeable decrease in the 0–0 transition band accompanied by ~ 8 nm hypsochromic shift is observed, which indicates that the aggregation become more pronounced in the neutral pH. At the basic medium (pH 9), a similar behavior was observed as in the natural solution where the absorption maximum corresponding to the 0–1 transition was recorded at 501 nm. However, a shoulder can still be observed at 534 nm indicating the 0–0 transition. In conclusion, the ratio of A_(0–0)_/A_(0–1)_ for the two main absorption bands of TAIPDI at acidic pH (pH = 4) (A_542 nm_/A_505 nm_ = 1.01) is much smaller than that observed in neutral pH, where (A_534 nm_/A_501 nm_ = 1.68), and basic pH (pH = 9), where (A_534 nm_/A_501 nm_ = 1.75), which means that the vibronic signature of PDI aggregates gets clearer by increasing pH values.

Similarly, the steady-state emission spectra using 480 nm excitation light (Fig. 7b) showed a systematic fluorescence quenching upon increasing the pH values. At acidic pH we can notice an emission maximum at 557 nm and another band at 599 nm corresponding to 0–0 and 0–1 transitions respectively. At neutral and basic pH, the emission maxima were found to be 549 and 591 nm corresponding to 0–0 and 0–1 transitions, respectively. From this finding, one can see ~ 8 nm hypsochromic shift between the emission maxima accompanied with a huge decrease in fluorescence intensity (4–5 folds approximately compared to acidic pH).

### Optical Properties of BSSBP in an Aqueous Medium

**Fig. 8:**
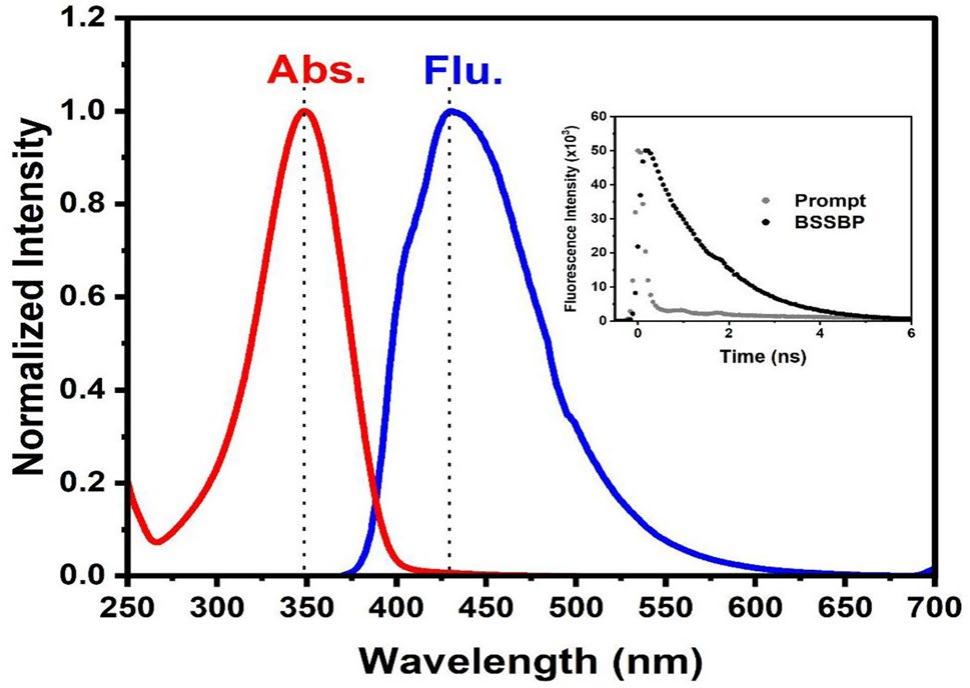
Steady-state absorption and fluorescence spectra of BSSBP in water; λ_ex_ = 350 nm. Inset: Decay time profile of BSSBP at 431 nm using laser diode head at 375 nm

The absorption spectrum of BSSBP (2 μM) exhibited abroad absorption band with a maximum absorption at 350 nm (Fig. 8). By using 350 nm excitation light, the fluorescence spectrum of BSSBP (5 μM) exhibited a strong fluorescence band with maximum fluorescence at 431 nm. Based on the values of absorption and emission maxima, the energy of the singlet excited state of BSSBP in an aqueous medium has been found to be 2.75 eV, which is higher compared to that of the singlet excited state of TAIPDI (2.25 eV). By using the TCSPC technique, the fluorescence lifetime of the singlet excited state of BSSBP was found to be 1.34 ns, which is much shorter compared to that of TAIPDI (4.75 ns).

### Spectroscopic Studies on TAIPDI@BSSBP Supramolecular Complex

**Fig. 9:**
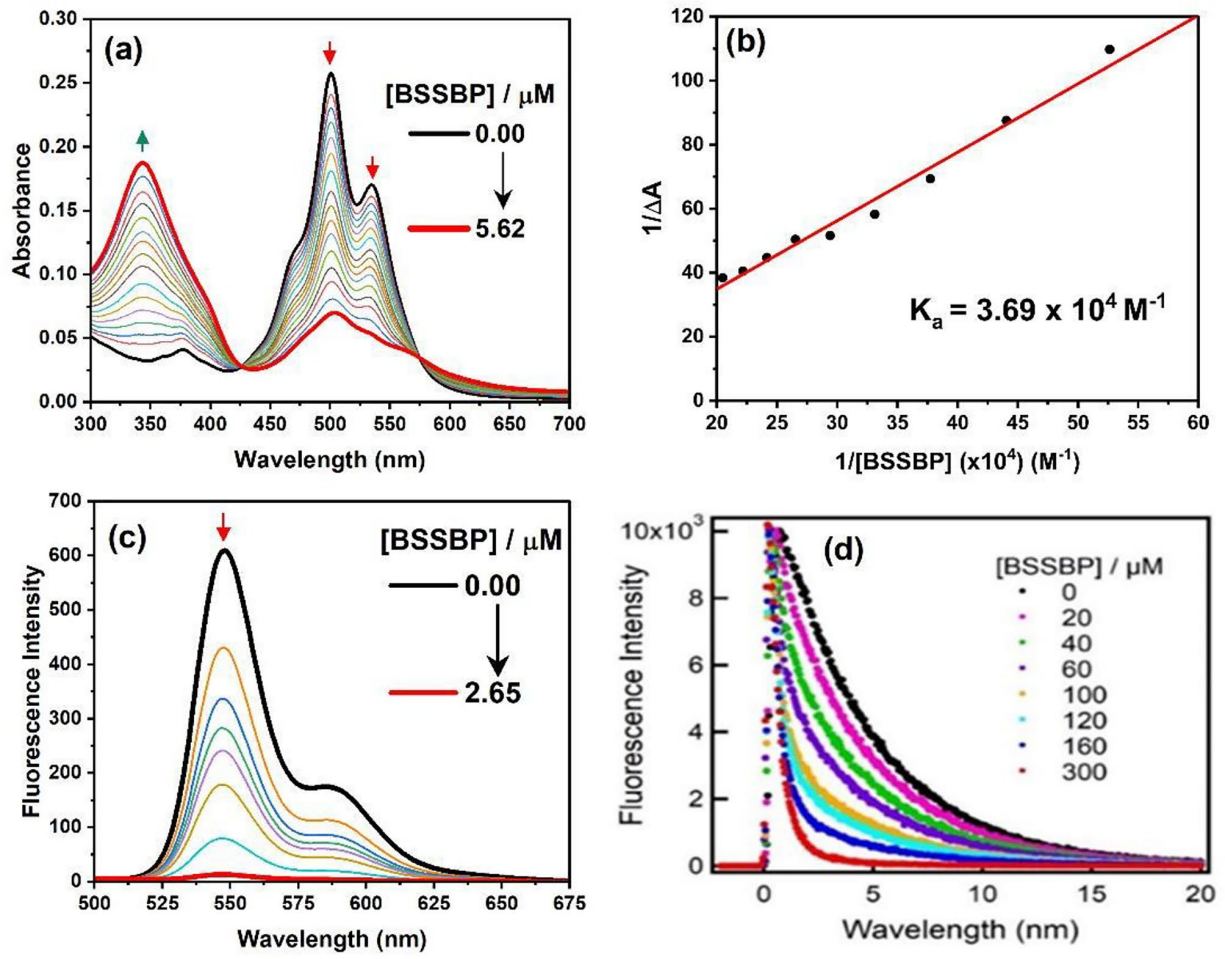
**a** Spectral changes in the absorption spectrum of TAIPDI (4.6 µM) during the titration with different concentrations of BSSBP in water at 25 °C, **b** the Benesi-Hildenbrand plot to determine the binding constant, **c** fluorescence spectral changes of TAIPDI (4.6 µM) during titration with BSSBP in water; λ_ex_ = 433 nm, **d** fluorescence decay profiles of TAIPDI in the presence of BSSBP in water; λ_ex_ = 440 nm and λ_*em*_ = 550 nm

In order to investigate the self-assembly formation between TAIPDI and BSSBP units, both UV–vis absorption and fluorescence measurements were carried out in an aqueous solution. As previously mentioned, the absorption spectra of TAIPDI (4.6 μM) in water show three pronounced absorption peaks at 540, 503, and 472 nm corresponding to 0–0, 0–1, and 0–2 transitions, respectively. Photometric titration of TAIPDI with different concentrations of BSSBP results in a decrease in the absorption feature bands of TAIPDI accompanied by a slight red-shift (~ 3 nm) and recording two clean isosbestic points at 424 and 585 nm (Fig. 9a). This finding indicates the formation of self-assembly between TAIPDI and BSSBP through strong ionic and π-π interaction. A plot of absorbance of the 501 nm band vs the number of equivalents of BSSBP (Fig. S4) revealed a break at around 1.3, which is expected for the 1:1 stiochiometery for complex formation between BSSBP and TAIPDI. Based on the Benesi-Hildebrand plot (Fig. 9b) [42]^1^, the binding constant (*K)* of the supramolecular TAIPDI-BSSBP complex was determined to be 3.69 × 10^4^ M^−1^, suggesting a moderately stable complex.

The intra-supramolecular complex formation between TAIPDI and BSSBP was further studied using steady-state fluorescence measurements. Upon photoexcitation of TAIPDI at 470 nm, three emission peaks were observed at 550, 590, and 645 nm which are mirror images of the absorption spectrum. When TAIPDI was titrated with different concentrations of BSSBP, a significant quenching of the emission peaks of TAIPDI occurred as shown in Fig. 9c. This quenching is most likely arising from the electron transfer from the ground state of BSSBP to the singlet excited state of TAIPDI, taking into account that the energy transfers from the singlet state of TAIPDI (2.25 eV) to the singlet of BSSBP (2.76 eV) in thermodynamically unfavorable.

The fluorescence lifetime measurements track the above steady-state fluorescence consideration in a more quantitative way, giving the rate and efficiency of the electron transfer process in water. As seen from Fig. 9d, the time profile of the singlet excited state of TAIPDI control in aqueous medium exhibited a single exponential decay with a lifetime of 4.30 ns. With the addition of different amounts of BSSBP to the solutions of TAIPDI in water, the decay profiles of the singlet excited states of TAIPDI get significantly accelerated. It is clear that the decay profile of TAIPDI-BSSBP satisfactorily fitted as bi-exponential decay; one has a short lifetime of 0.21 ns which reflects the actual intramolecular deactivation of the singlet TAIPDI, and the other has a larger lifetime of 3.87 ns, which is close to the TAIPDI reference. Based on the lifetime of the singlet excited state of TAIPDI reference (τ_0_) and the fast decay of the TAIPDI-BSSBP complex (τ_f_), the rate (*k*_et_) and quantum yield (φ_et_) of the electron transfer process were determined to be 4.76 × 10^9^ s^−1^ and 0.95, respectively [43–45]^2^. These values suggest fast and efficient electron transfer from the ground state BSSBP to the singlet excited state of TAIPDI.

**Fig. 10:**
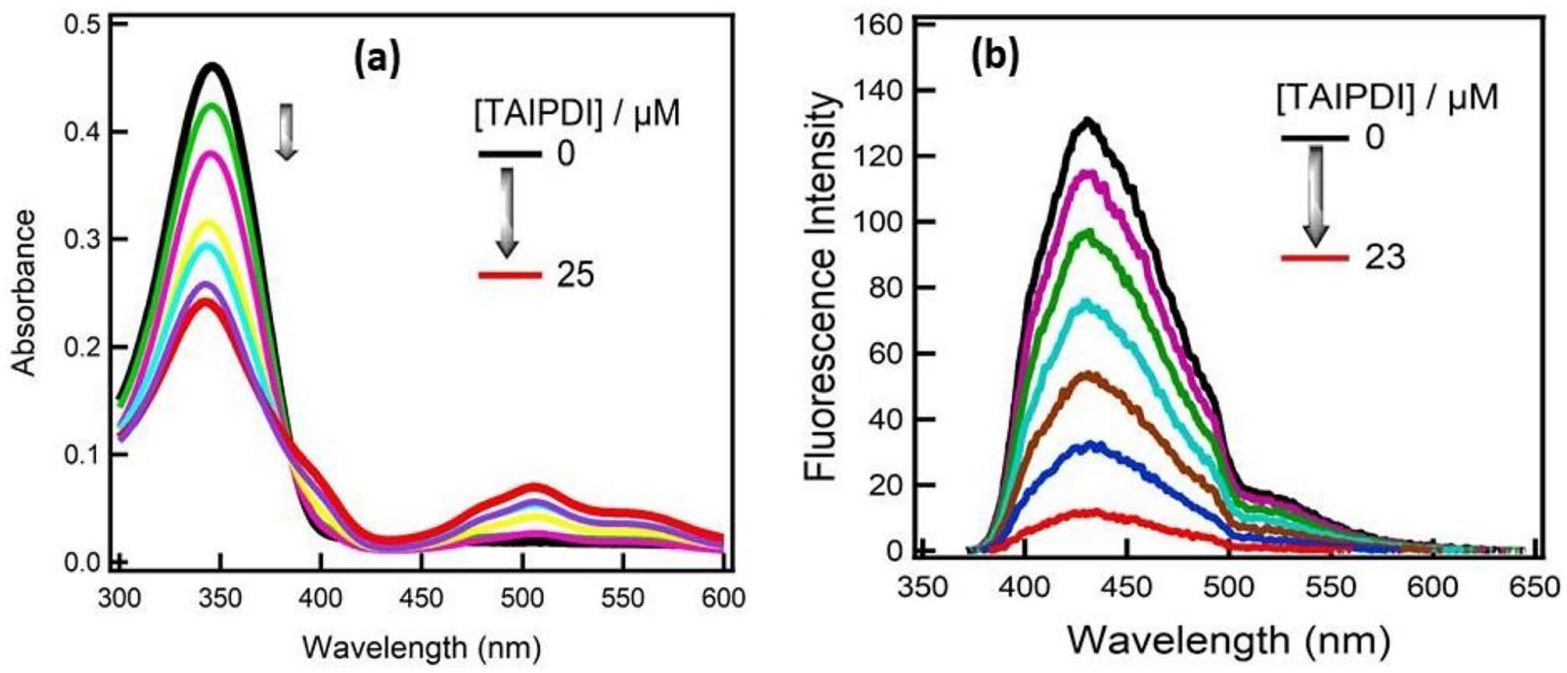
**a** Absorption spectral change during the titration of BSSBP (20 μM) with different concentrations of TAIPDI (0–25 μM) in water, **b** fluorescence spectral change during the titration of BSSBP with different concentrations of TAIPDI (0–23 μM) in water

It is worth mentioning that a similar electron transfer character was also observed upon adding different amounts of TAIPDI (up to 25 μM) to an aqueous solution of BSSBP. Figure 10a. showed a considerable decrease in the absorbance of the BSSBP entity at 350 nm accompanied by an increase in the absorbance of the TAIPDI entity at 550 nm. In addition, a significant quenching of the fluorescence intensity of BSSBP at 450 nm was recorded in Fig. 10b by adding various amounts of TAIPDI (up to 23 μM). These findings confirm the electron transfer process from the singlet excited state of BSSBP (2.76 eV) to the non-covalently linked TAIPDI in the supramolecular dyad.

The observed electron transfer character from BSSBP to TAIPDI in the formed supramolecular complex has been supported by the electrochemical measurements of BSSBP and TAIPDI entities in water was recorded as shown in Fig. S5. The first two reduction potentials (E_red_) of TAIPDI were recorded as -0.52 and -0.84 V *vs.* Ag/AgCl, while the first oxidation potential (E_ox_) of BSSBP was recorded as 0.74 V *vs.* Ag/AgCl. Based on the first reduction potential of TAIPDI and the first oxidation potential of BSSBP, the energy of the electron transfer product was calculated to be 1.26 eV. Based on the energy of the singlet excited state of TAIPDI (2.25 eV) and BSSBP (2.76 eV), the driving forces of the electron transfer (-ΔG_et_) via the singlet excited state of TAIPDI and BSSBP were calculated to be 0.99 and 1.50 eV, respectively [44]. Such negative ΔG_et_ of the radical ions pair indicates an exergonic electron transfer via both singlet excited states of TAIPDI and BSSBP.

### Computational Studies

**Fig. 11:**
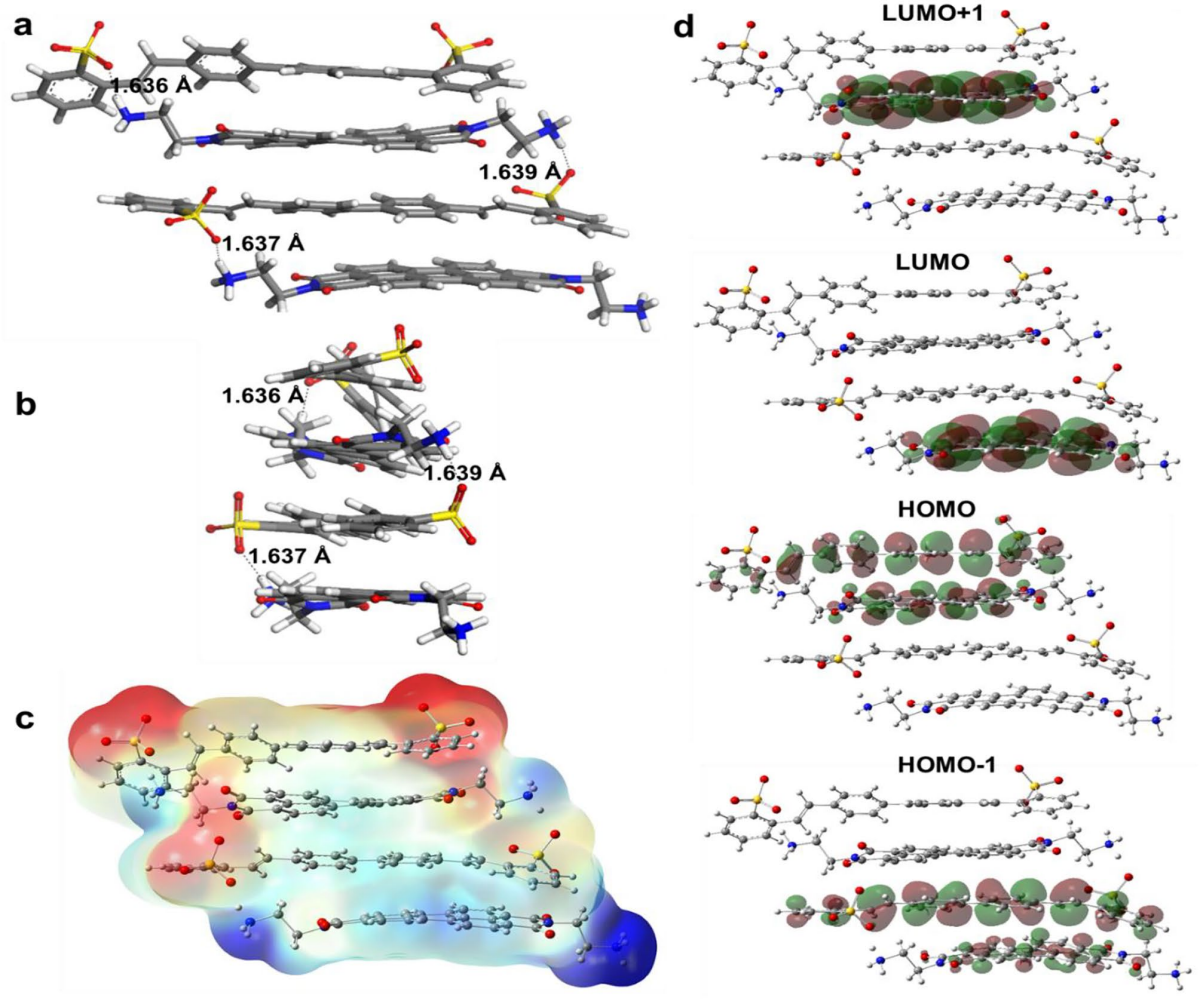
**a**, **b** Geometry optimized structure for the sequential aggregation of BSSBP and TAIPDI, **c** ESP surface for the aggregated complex, **d** Isosurface for the HOMO-1, HOMO, LUMO, and much attention LUMO + 1 orbitals

The lowest energy structure for the quaternary aggregate given in Fig. 11a, b shows that there are both ionic interactions between the anionic sulfonate group of BSSBP and cationic trimethyl ammonium group of TAIPDI, as well as π-stacking between both entities [45–49]. Due to the strong ionic interactions of the side groups that bring the ionic end groups as close as 1.64 Å distance in water and π-stacking distances were decreased to as low as 3.31 Å, which is slightly shorter than the conventional π-stacking suggesting the substantial charge transfer and the strong tendency for aggregation. The electrostatic potential surface shown in Fig. 11c demonstrates consecutive electron-deficient groups on TAIPDI and electron-rich groups at the ionic end groups and a relatively neutral π-stacked middle part that leads to the exceptional molecular self-organization, self-assembly, and electronic structure due to this packing motif in water. The close intermolecular distance and self-assembly between the electron accepting TAIPDI and the electron donating BSSBP were also depending on the similar size of the BSSBP and TAIPDI having 22–23 Å end distance.

Electronic structure calculated by M06-2X functional shows that occupied frontier molecular orbitals HOMO and HOMO-1 are located on the BSSBP, while the LUMO and LUMO + 1 were located on the TAIPDI, which indicates a formation of a donor–acceptor complex in water (Fig. 11d). Charge transfers per one acceptor and one donor unit were calculated as 0.5, 0.2 and 0.2 e^−^ by CM5, ESP, and natural population analysis charge calculation algorithms [50], respectively. CM5 calculation that depends on the molecular dipole moment as the key quantity, that the mapped charges were set to reproduce this dipole, is more reliable for this type of polar system. Other functionals gave close results for the same charge algorithms and orbital surfaces. These charge transfers are very high values per molecule for this kind of charge transfer complexes in solution that can be exploited for the preparation of nanowires with enhanced optoelectronic properties. The interaction energy per BSSBP^2−^ and TAIPDI^2+^ were calculated as 3.57 eV in the perfect optimized packing structure in implicit water.

**Fig. 12:**
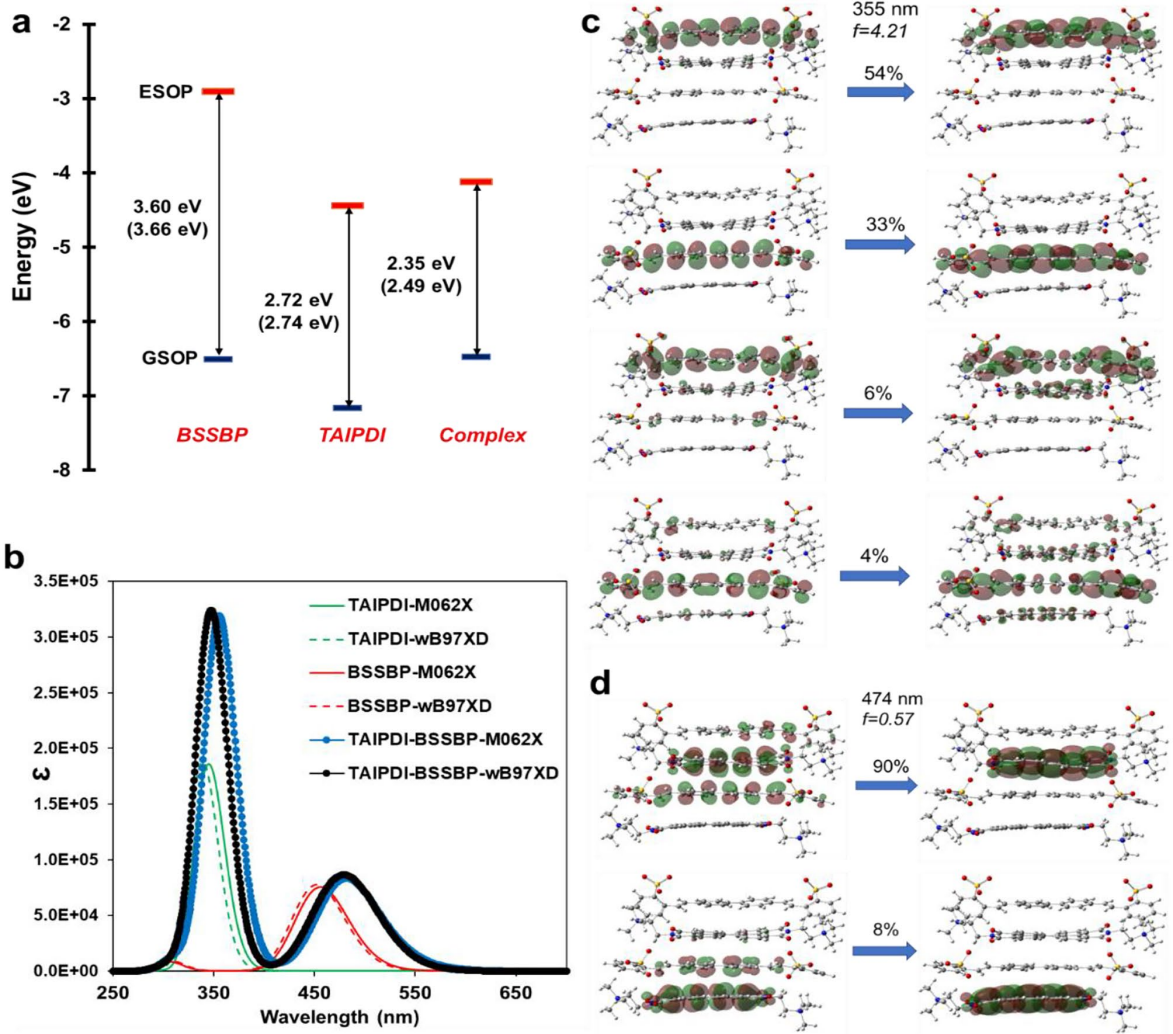
**a** GSOP, ESOP energy levels, and band gap values for BSSBP, TAIPDI, and complex by M06-2X method using wB97XD energy functional with 6-31 g(d) basis sets; results are given in parenthesis, **b** UV spectra for the BSSBP anion, TAIPDI cation, aggregated complex, **c**–**d** Natural transition orbitals and their probabilities for the two main UV absorption peak of the aggregated complex

Energy levels for the ground state and excited state oxidation potentials (GSOP and ESOP) and band gap (*E*_*0-0*_) point out that GSOP for the aggregated charge transfer complex has a close value with the GSOP of the BSSBP donor, and ESOP for the aggregated charge transfer complex has a close value with the ESOP of the TAIPDI acceptor, resulting in the decreased band gap (Fig. 12a). The difference between the frontier orbital energy levels of individual molecules and aggregated complex points out a strong charge transfer and coupling between the BSSBP and TAIPDI. UV spectra for BSSBP, TAIPDI, and complex are given in Fig. 12b show the broad absorption peak for the complex and demonstrate potential control of the UV absorption spectra by band gap engineering and charge transfer based on the packing structure by tailoring the aromatic structures in the center as well as charged anion and cation end groups that are responsible for the solvation. Calculated absorption spectra have close values to the experimental spectra given in Fig. 4. A broad shoulder at approximately 467 nm was determined at 455 nm in theoretical calculations for TAIPDI and a broad peak started from 300 nm was reproduced for BSSBP. A maximum absorption peak at 355 nm in water due to $\uppi -\uppi$* electronic transitions was calculated for the complex that was experimentally determined at 350 nm. The absorption peaks of TAIPDI increased accompanied by a bathochromic shift up to 10 nm were perfectly reproduced by DFT calculations that validate the proposed packing motif for the TAIPDI-BSSBP complex. Close intermolecular distances and symmetric packing indicate the formation of a highly stable self-assembled complex in water leading to higher electron transfer values and improved control of the UV–Vis absorption. Two important absorption peaks with the highest oscillatory frequencies were characterized by NTOs given in Fig. 12c, d, demonstrating that, in addition to the transitions observed for TAIPDI and BSSBP, charge transfer contributions were present at different probabilities which are responsible for the experimentally observed peak shifts.

## Conclusion

The optical behavior of TAIPDI has been examined in different solvents using various spectroscopic techniques. While TAIPDI is present in its monomer form in organic solvents, the TAIPDI molecule in water has proven effective for π-π stacking and interactions between the aromatic centers owing to the minimal side-chain steric hindrance, forming one-dimensional (1D) nanostructure. By examine the effect of concentrations and temperatures, it was concluded that the high concentrations of TAIPDI and/or low temperatures would result in closer packing of TAIPDI chromophores as the exciton coupling grows stronger, favoring the aggregation behavior of TAIPDI in water. In order to control the aggregation behavior of TAIPDI in water, which is of great importance for various applications, the optical properties of TAIPDI have been examined in the presence of cetyltrimethylammonium bromide (CTAB), and sodium dodecyl sulfate (SDS). While there was no significant effect for the addition of CTAB, the addition of various amounts of SDS caused a significant effect in both the absorption and emission spectra, from which the CMC values were found to be 0.03 and 0.04 M, respectively. The photo-driven intra-supramolecular electron transfer of the light-harvesting TAIPDI-BSSBP complex has been examined using various spectroscopic techniques. Based on the absorption measurement, the formation constant of the supramolecular complex was found to be 3.69 × 10^4^ M^−1^, suggesting a moderately stable complex. The steady-state and time-resolved fluorescence measurements showed a significant emission quenching of the singlet excited state of TAIPDI with the addition of various amounts of BSSBP in water suggesting the electron transfers from BSSBP to the electron-accepting TAIPDI with a rate constant and efficiency of 4.76 × 10^9^ s^−1^ and 0.95, respectively. A similar electron transfer character was observed from the singlet excited state of BSSBP to its supramolecularly-linked TAIPDI. The ease of construction, strong absorption in the visible region, and fast and efficient electron transfer process render the supramolecular TAIPDI-BSSBP complex a promising light-harvesting material for converting photonic energy into chemical energy.

### Supplementary Information

Below is the link to the electronic supplementary material.

Supplementary file1 (DOCX 986 KB)

## Data Availability

The authors confirm that the data supproting the findings of this study are available within the article. Raw data supporting the findings of this study are available from the corresponding author upon reasonable request.

## Footnotes

1 The binding constant *K* for the complex formation between TAIPDI and BSSBP was determined from the absorption spectral data by using the Benesi-Hildebrand method according to the following equation: $\frac{1}{A-A0}= \frac{1}{(Kb*\left(Amax-A0\right)*\left[BSSBP\right])}+\frac{1}{Amax-A0}$ Here A_0_ is the absorbance of TAIPDI in the absence of BSSBP, A is the absorbance recorded in the presence of added BSSBP, A_max_ is the absorbance in presence of added [BSSBP]_max_ and K_b_ is the association constant. From the plot of 1/(A-A_0_) against 1/[BSSBP] shown in Fig. 9b, the binding constant of the supramolecular complex TAIPDI-BSSBP was found to be 3.69 × 10^4^ M^-1^

2 Based on the lifetimes of the singlet excited states of TAIPDI reference (τ_0_) and the fast decay of the TAIPDI-BSSBP complex (τ_f_), the rate of the electron transfers process (-*k*_et_) and quantum yield (Φ_et_) were calculated from the following equations: ${K}_{et}={(1/{\tau }_{\mathrm{f}0})}_{\mathrm{sample}}-{(1/{\tau }_{\mathrm{f}})}_{\mathrm{reference}}$ ${\Phi }_{\mathrm{et}}={k}_{et}/{(1/{\tau }_{f})}_{\mathrm{sample}}$
