# Supplementary material for: Optical Properties of Cationic Perylenediimide Nanowires in Aqueous Medium: Experimental and Computational Studies
Source: J Fluoresc. 2023 Jun 6;34(1):411–24. doi: 10.1007/s10895-023-03253-9 (PMC10808178; doi:10.1007/s10895-023-03253-9)
Supplement: Supplementary file 1 — Supplementary file1 (DOCX 986 KB) [file 10895_2023_3253_MOESM1_ESM.docx]

**Supplementary information’s**


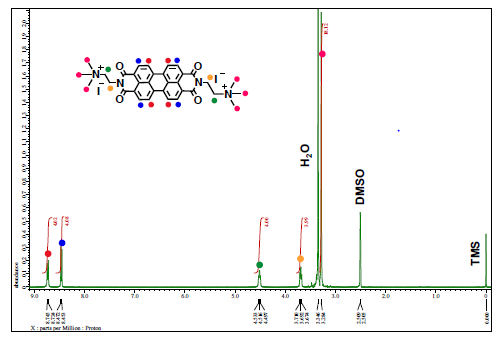

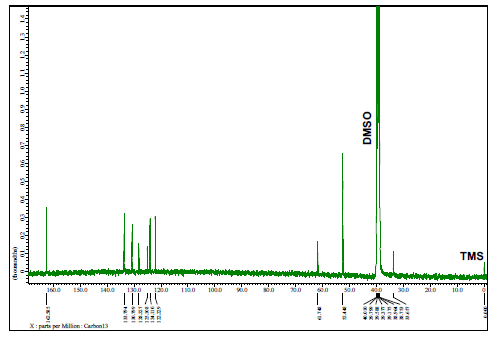


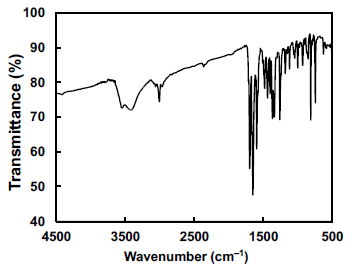

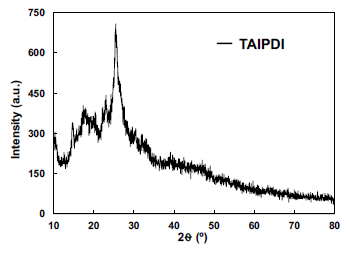


**Fig. S1** (a) ^1^H NMR of TAIPDI in DMSO-d6, (b) ^13^C NMR of TAIPDI in DMSO-d6, (c) FT-IR spectra of TAIPDI on a KBr plate, and (d) XRD pattern of TAIPDI.


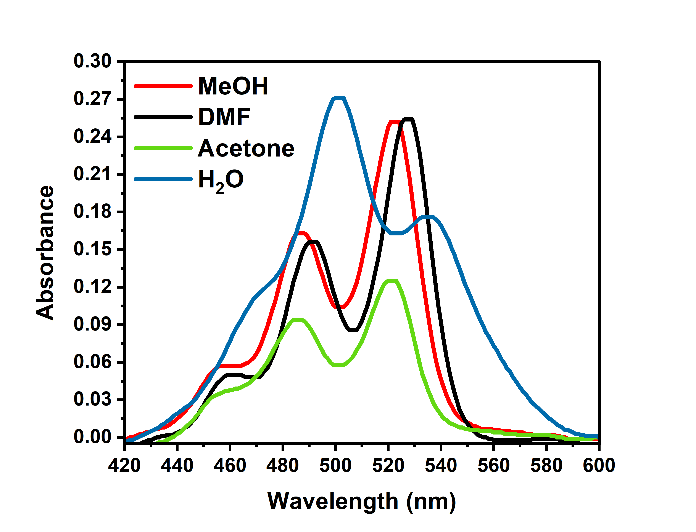

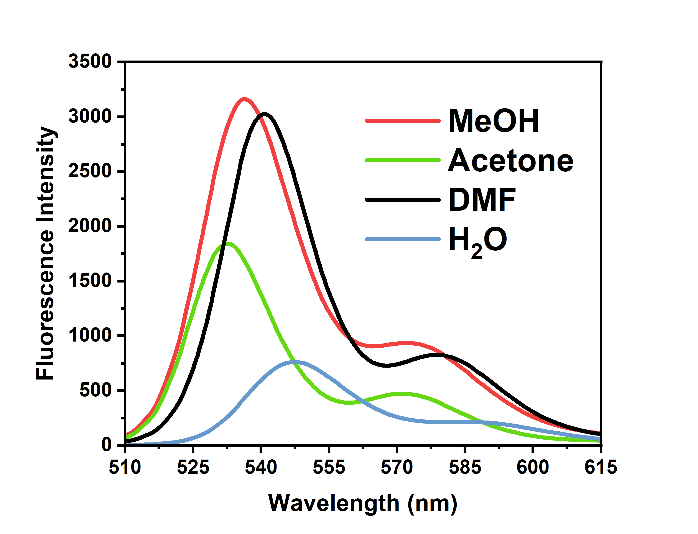

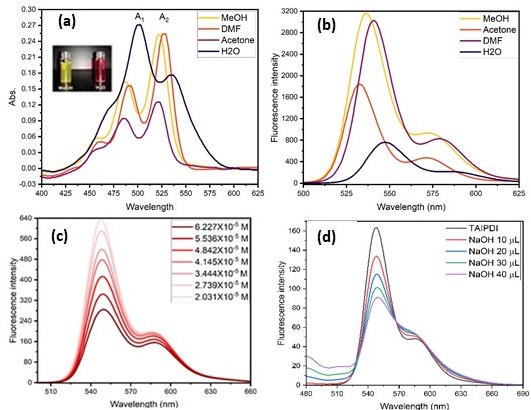


**(a)**

**(b)**

**Fig. S2** Steady-state absorption (a) and fluorescence (b) of TAIPDI in different solvents (H_2_O, MeOH, DMF, and acetone).

**
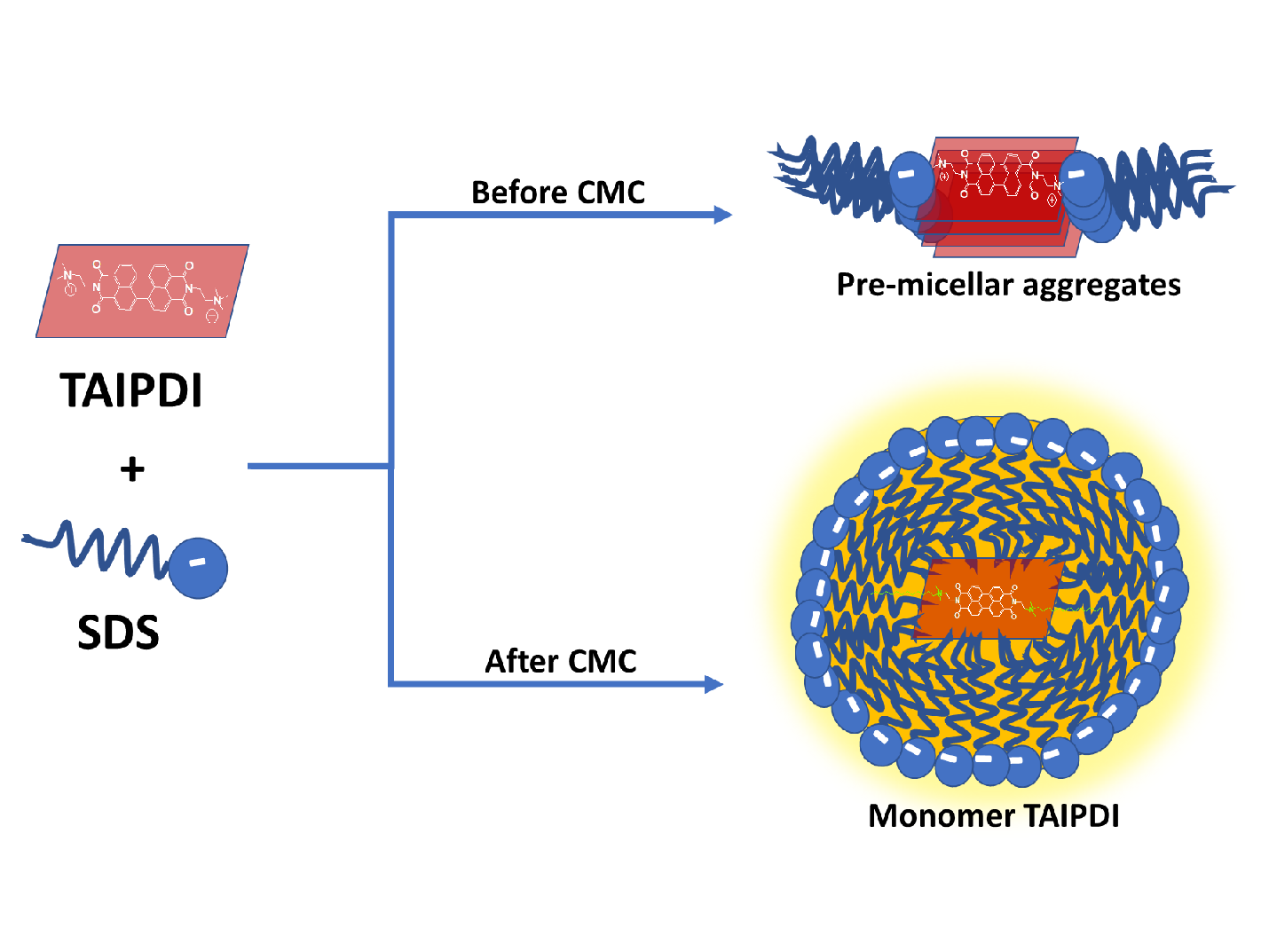
**

**Fig. S3** Graphical representation of the proposed mechanism of the fluorescence behavior upon adding different concentrations of SDS to TAIPDI.

**Fig. S4 Plot of the absorbance change at 501 nm vs the ratio of [BSSBP]/[TAIPDI].**

***
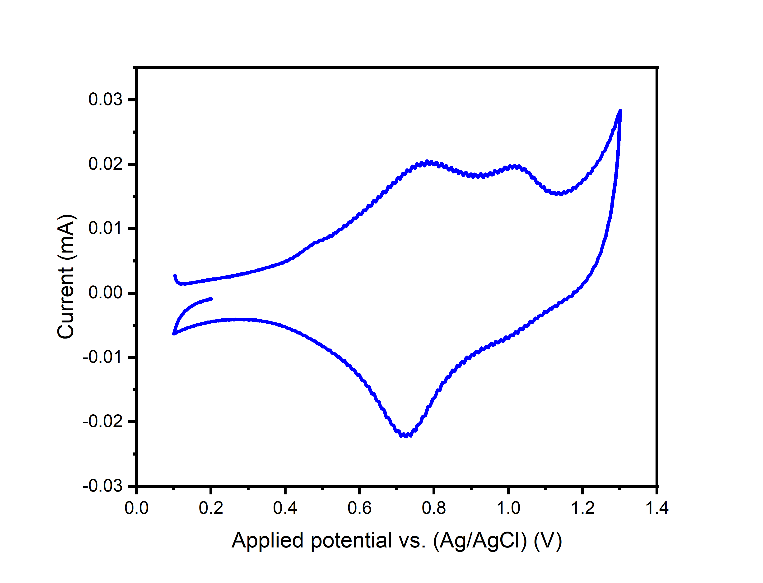
***

**Fig. S5** Cyclic voltammograms of TAIPDI (upper figure) and BSSBP (lower figure) in water containing Na_2_SO_4_ (0.1 M). Scan rate = 20 mV s^-1^.
